# Lung CCR6^−^CXCR3^−^ type 2 helper T cells as an indicator of progressive fibrosing interstitial lung diseases

**DOI:** 10.1038/s41598-022-24011-0

**Published:** 2022-11-15

**Authors:** Tsukie Kin Tsukuda, Hiroshi Ohnishi, Minoru Fujimoto, Yu Nakatani, Kazufumi Takamatsu, Tetsuji Naka, Akihito Yokoyama

**Affiliations:** 1 Department of Respiratory Medicine and Allergology, Kochi Medical School, Kochi University, Kohasu, Oko-Cho, Nankoku, Kochi 783-8505 Japan; 2 Center for the Intractable Immune Disease, Kochi Medical School, Kochi University, Nankoku, Japan; 3 Division of Allergy and Rheumatology, Department of Internal Medicine, School of Medicine, Iwate Medical University, Morioka, Japan

**Keywords:** Respiratory tract diseases, Adaptive immunity

## Abstract

Progressive fibrosing interstitial lung diseases (PF-ILDs) have a poor prognosis and may be resistant to corticosteroids and/or immunosuppressants, but antifibrotic therapies such as nintedanib and pirfenidone have been shown to slow the deterioration of lung function. The aim of this study was to identify the characteristic cellular profile of bronchoalveolar lavage fluid at diagnostic bronchoscopy for predicting PF-ILDs, defined as fibrotic diseases on chest high-resolution computed tomography with more than a 5% relative decline in the percent predicted value of forced vital capacity (FVC) over 6 months. The proportions of inflammatory cells, CCR6^−^CXCR3^−^ T helper type 2 (Th2) cells among conventional CD4^+^ T cells in bronchoalveolar lavage fluid (BALF) and peripheral blood, were measured by flowcytometry. The proportion of lymphocytes in BALF was significantly higher in non-PF-ILD patients than in PF-ILD patients. The proportion of Th2 cells in BALF, but not in peripheral blood, was significantly higher in PF-ILD patients than in non-PF-ILD patients. Multivariate analysis showed that a greater population of Th2 cells in BALF was the only indicator for PF-ILDs. An increased proportion of Th2 cells in BALF is associated with greater deterioration of lung function in fibrotic interstitial lung diseases.

## Introduction

Interstitial lung diseases (ILDs) consist of more than 200 diffuse lung diseases, most of which are associated with pulmonary fibrosis. Idiopathic pulmonary fibrosis (IPF) is classified as an idiopathic interstitial pneumonia (IIP) with the most frequent progressive fibrosing phenotype and the worst prognosis. Other ILDs such as idiopathic non-specific interstitial pneumonia (NSIP), connective tissue disease-associated ILD (CTD-ILD), unclassifiable IIPs, fibrotic hypersensitivity pneumonitis (HP), and pneumoconiosis often show similar fibrotic clinical features: progression of pulmonary fibrosis, worsening exertional dyspnea, progressive decline in lung function, resistance to corticosteroids and/or immunosuppressive therapies, and poor prognosis^1–4^. They are categorized as progressive fibrosing interstitial lung diseases (PF-ILDs) or ILDs with a progressive fibrosing phenotype, and antifibrotic therapies such as nintedanib and pirfenidone have been shown to improve disease progression of PF-ILDs, including IPF, CTD-ILD, and fibrotic HP^5–7^.

Prediction of future deterioration of lung function and/or progressive pulmonary fibrosis is difficult in real-world clinical settings, especially in patients with non-usual interstitial pneumonia (UIP) pattern on chest high-resolution computed tomography (HRCT), even though imaging resolution has improved remarkably. In such patients, sequential changes in pulmonary fibrosis on chest HRCT and/or in forced vital capacity (FVC) are often evaluated to determine whether antifibrotic therapy should be started, but this follow-up may delay the treatment of PF-ILDs. Identifying PF-ILD at diagnosis is extremely important for improving patients’ prognosis.

There is an urgent and unmet need to identify predictors of responsiveness to corticosteroids, immunosuppressants, and/or antifibrotic drugs. The underlying pathological mechanism of fibrosis in PF-ILDs remains unclear and can be complicated by the involvement of multiple factors. Bronchoalveolar lavage fluid (BALF) containing various immune cells recovered from the lungs is the best research sample to identify such a mechanism. In the present study, cellular profiles of T cell subsets were compared in BALF and the peripheral blood of patients with fibrotic ILDs using multicolor flow cytometry, and a characteristic cellular profile of helper T (Th) cells in BALF to predict PF-ILDs was found.

## Methods

### Patient population and collection of samples

Patients with fibrotic ILDs (traction bronchiectasis, architectural distortion, and/or honeycombing on chest HRCT) who underwent bronchoscopy in our hospital from April 24, 2019 to December 24, 2021 were enrolled in this study [n = 30, IPF (n = 9), HP (n = 11), and CTD-ILD (n = 10)]. Diagnoses of IPF and HP were based on the Official ATS/ERS/JRS/ALAT Clinical Practice Guideline and the Official ATS/JRS/ALAT Clinical Practice Guideline, respectively^8,9^. The diagnosis of CTD-ILD was based on the presence of ILD on chest HRCT and the common diagnostic criteria for each respective CTD^10–13^.

Participants underwent chest HRCT before bronchoscopy. BALF (n = 30) and peripheral blood (n = 29) were obtained from these patients on the same day or within 1 day. BALF cell differentiation was assessed by morphologically counting at least 400 cells on cytospin slides after May-Giemsa staining. Serum Krebs von den Lungen-6 (KL-6) levels were measured by the chemiluminescent enzyme immunoassay LUMIPULSE KL-6 using a KL-6 antibody (SEKISUI MEDICAL Co., Ltd., Tokyo, Japan). The cut-off value for serum KL-6 was set at 435 U/ml according to the manufacturer’s instructions. Patients with coexisting active respiratory infections, patients being treated with antifibrotic drugs and/or anticancer agents including immune checkpoint inhibitors, and patients with insufficient BALF recovery (recovery rate less than 35%) were excluded.

This study was approved by the ethics committee of the Kochi Medical School, Kochi University (Approval number 202-102) and was registered in the UMIN Clinical Trials Registry (Registry ID UMIN000041871). Written, informed consent was obtained from all participants. This study complied with the Declaration of Helsinki.

### Interpretation of chest HRCT images

Chest HRCT findings were reviewed separately by two independent respiratory specialists who were not aware of the patients’ profiles. The presence of traction bronchiectasis, architectural distortion, and/or honeycombing on chest HRCT provided evidence of lung fibrosis based on the guideline of the Fleischner Society. Cases with traction bronchiectasis and/or architectural distortion without honeycombing were further defined as fibrotic NSIP (f-NSIP) patterns, whereas cases with honeycombing were defined as a UIP pattern. Following the initial independent chest HRCT assessment, discordant evaluations were resolved by consensus reached after consultation between the two observers.

### Flow cytometry

**Figure 1:**
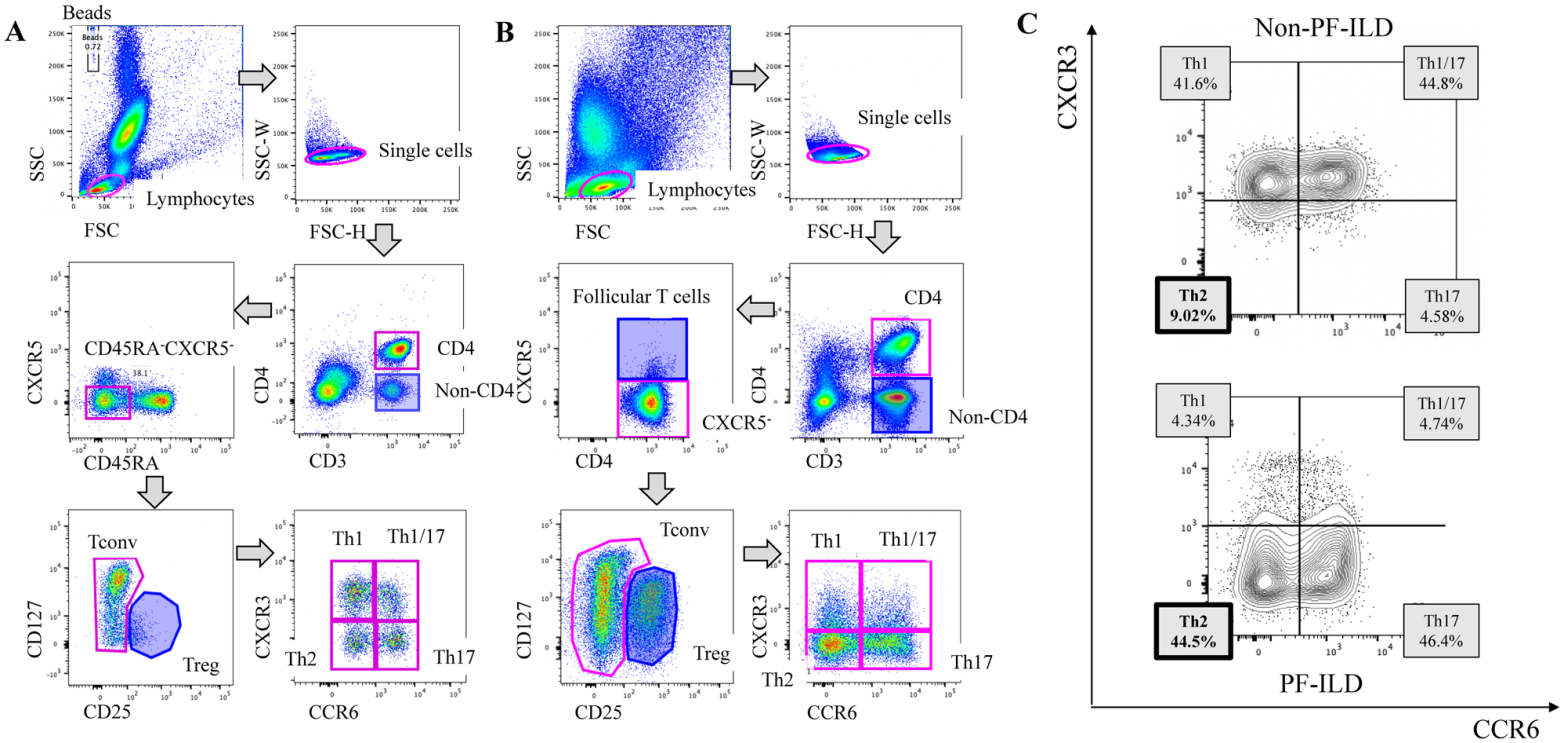
Gating strategy of flow cytometry for identifying helper T cell (Th) subsets. For analysis of peripheral blood (**A**), we gated on live lymphocytes using forward scatter (FSC) and side scatter (SSC), followed by exclusion of doublets, then identified CD3^+^CD4^+^ T cells. Among these CD3^+^CD4^+^ T cells, we gated on CD45RA^−^CXCR5^−^ populations to gate out CD45RA^+^ naïve T cells and CXCR5^+^ follicular helper T cells, then gated on CD127^Low^ or CD127^High^ conventional T cells (Tconv) and to gate out regulatory T cells (Treg) using CD25 and CD127. CCR6 and CXCR3 for the classification of Th subsets (refer to Table 1). The same gating strategy was applied for analysis of BALF cells (**B**). (**A**, **B**) are examples of mixed connective tissue disease-associated ILD. Representative data on the differences in Th cell subset of BALF (**C**) between two IPF patients with non-PF-ILD phenotype (upper) or PF-ILD phenotype (lower). The proportion of Th2 cells are defined by the expression of surface markers CD4^+^CD45RA^−^CXCR5^−^CD25^low〜intermediate^CCR6^−^CXCR3^−^. *BALF* bronchoalveolar lavage fluid, *FSC* forward scatter, *ILD* interstitial lung disease, *PF-ILD* progressive fibrosing interstitial lung disease, *SSC* side scatter, *Tconv* conventional T cells, *Treg* regulatory T cells, *Th1 cell* T helper type 1 cell, *Th17 cell* T helper type 17 cell, *Th1/17 cell* Th1/Th17 hybrid cell, *Th2 cell* T helper type 2 cell.

For staining of cell surface markers, cells in BALF and whole blood were incubated with the following fluorescent-conjugated anti-human antibodies (CD25-FITC, clone BC96; CD185-PerCP-Cy5.5, clone J252D4; CD127-PE-Cy7, clone A019D5; CD3-APC-Cy7, clone UCHT1; CD4-BV510, clone OKT4; CD25-BV605, cloneBC96; HLA-DR-PE-Dazzle594, clone L243; CD45RA-BV421, clone HI100; CD38-BV605, clone HIT2; CD38-FITC, clone HIT2 [BioLegend, San Diego, CA]; CD196-PE, clone11A9; CD183-APC, cloneIC6/CXCR3; CCR4-BV421, clone1G1 [BD Pharmingen, San Jose, CA]). The online supplement provides the details of the procedure. The FACSAria Fusion and FACS LSRFortessa (BD Biosciences, San Jose, CA) and FlowJo software (Tree Star, Inc., Ashland, OR) were used for flow cytometric analyses. The gating strategy for identifying Th subsets is shown in Fig. 1 [peripheral blood (A) and BALF (B)]. Classification of Th subsets (Th1, Th1/17, Th17, and Th2) among conventional CD4^+^ T cells by CCR6 and CXCR3 expressions is shown in Table 1^14^.

**Table 1:** Definition of helper T cell subsets based on the surface expression of chemokine receptors.

	CCR6	CXCR3
Th1	−	+
Th1/17	+	+
Th17	+	−
Th2	−	−

*Th1* T helper type 1, *Th17* T helper type 17, *Th1/17* Th1/Th17 hybrid, *Th2* T helper type 2.

### Pulmonary function tests and definition of PF-ILDs

Pulmonary function tests were performed 6 months before bronchoscopy as a baseline and a second time within 1 month before bronchoscopy in 18 cases, or within 1 month before bronchoscopy as a baseline and a second time 6 months after bronchoscopy in 12 cases. Intervals of pulmonary function tests within 1 month were allowed. PF-ILDs were defined as having both radiological progression of chest shadows on chest HRCT and a relative percent predicted value of forced vital capacity (%FVC) decrease of 5% or more over 6 months despite disease management deemed appropriate by the physician (e.g., antigen avoidance and treatment with corticosteroids ± immunosuppressive agents) in this study. An improvement or worsening of %FVC was defined as an increase or decrease in relative %FVC change over 6 months.

### Statistical analysis

Data are presented as medians (interquartile range [IQR]). Differences between two groups were evaluated by the Mann–Whitney U test. The correlation coefficients between two variables were calculated using Spearman’s rank correlation coefficient (Rs). Quantitative differences were tested by the Chi-squared test for goodness of fit or by Fisher’s exact test. Interobserver agreement for chest HRCT findings was assessed using Kappa statistics. Kappa values greater than 0.61 were considered to indicate good agreement between observers. Multivariate analysis by binomial logistic regression was used to evaluate independent predictors associated with PF-ILDs. All statistical analyses were performed using IBM SPSS Statistics version 28 Standard (IBM Corp, NY, USA). Statistical significance was set at *p* < 0.05.

## Results

### Evaluation of Th cell subsets

Examples of Th cell subsets in a patient with mixed connective tissue disease-associated ILD are shown in Fig. 1A,B. The proportion of Th2 cells in peripheral blood (1A) and BALF (1B) was 18.9% and 50.9%, respectively. Representative data for the differences in Th cell subsets between two IPF patients with non-PF-ILD or PF-ILD phenotypes are shown in Fig. 1C. The proportion of Th2 cells, defined by the expression of surface markers CD4^+^CD45RA^−^CXCR5^−^CD25^low−intermediate^CCR6^−^CXCR3^−^, was increased in the BALF of patients with the PF-ILD phenotype (44.5%) compared to the non-PF-ILD phenotype (9.02%).

### Patients’ characteristics

**Figure 2:**
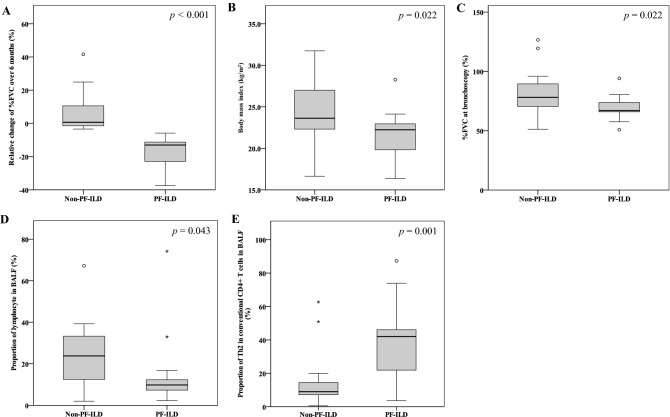
Comparison of variables between progressive fibrosing interstitial lung diseases (PF-ILDs) and non-PF-ILDs. (**A**) Relative change of %FVC over 6 months; (**B**) body mass index; (**C**) %FVC at bronchoscopy; (**D**) the proportion of lymphocytes in BALF; (**E**) proportion of Th2 cells among conventional CD4^+^ T cells in BALF. Differences between two groups were evaluated by the Mann–Whitney U test. *BALF* bronchoalveolar lavage fluid, *FVC* forced vital capacity, *PF-ILDs* progressive fibrosing interstitial lung diseases, *Th2 cells* T helper type 2 cell.

Of 30 patients with fibrotic ILDs, 13 (43.3%) were determined to have PF-ILDs with a relative %FVC decline of 5% or more over 6 months (Table 2). Patients with PF-ILDs had a 13.0% (IQR 11.6%) reduction in the median relative change in %FVC predicted over 6 months compared to 0.6% (IQR 12.0%) in patients with non-PF-ILDs (*p* < 0.001, Table 2, Fig. 2A). There were no differences in age, height, weight, sex, smoking status, serum KL-6 level, serum lactate dehydrogenase (LDH) level, diagnosis of ILD, or chest HRCT pattern between non-PF-ILDs and PF-ILDs at bronchoscopy (Table 2). The body mass index (BMI) and %FVC predicted at bronchoscopy were significantly lower in patients with PF-ILDs than in those with non-PF-ILDs (BMI 22.2 vs 23.6 kg/m^2^, *p* = 0.022, Fig. 2B; %FVC predicted 67.0% vs 78.1%, *p* = 0.022, Fig. 2C). On chest HRCT, 6 patients were classified as f-NSIP pattern and 24 as UIP pattern. Interobserver variability of chest HRCT findings before reaching consensus agreement, expressed as a Kappa value, was 0.74, indicating good agreement between two observers.

**Table 2:** Patients’ characteristics compared between the non-PF-ILD and PF-ILD groups.

	Non-PF-ILD n = 17 (56.7%)	PF-ILD n = 13 (43.3%)	P value
Age (year)	71 (22)	73 (7)	0.408
Height (cm)	160.5 (9.9)	159.6 (10.8)	0.621
Body weight (kg)	60.6 (18.5)	50.7 (12.3)	0.065
Body mass index (kg/m^2^)	23.6 (4.7)	22.2 (3.1)	0.022
Sex, male [n (%)]	10 (58.8)	10 (76.9)	0.44
Smoking status (n) Never/former/current	3/13/1	3/10/0	1.00
Serum KL-6 level (U/mL)	1248 (1278)	797 (735)	0.113
Serum LDH level (U/L)	238 (73)	241 (44)	1.000
**Diagnosis of ILD (n)**			0.588
IPF	4	5	
Fibrotic HP	6	5	
CTD-ILD	7	3	
SSc	3	1	
PM/DM	3	1	
SjS	0	1	
MCTD	1	0	
**Chest HRCT pattern (n)**			0.196
f-NSIP	5	1	
UIP	12	12	
%FVC predicted at diagnostic BF (%)	78.1 (19.0)	67.0 (7.8)	0.022
Relative %FVC change over 6 months (%)	0.6 (12.0)	-13.0 (11.6)	< 0.001
**Treatment during FVC measurements**			0.762
None	9	10	
PSL	3	1	
PSL + Tac	1	1	
Antigen avoidance	3	1	
Nintedanib	1	0	

*BF* bronchoscopy, *CTD* connective tissue disease, *f-NSIP* fibrotic nonspecific interstitial pneumonia, *FVC* forced vital capacity, *HP* hypersensitivity pneumonitis, *HRCT* high-resolution computed tomography, *ILD* interstitial lung disease, *IPF* idiopathic pulmonary fibrosis, *KL-6* Krebs von den Lungen-6, *LDH* lactate dehydrogenase, *MCTD* mixed connective tissue disease, *PF-ILD* progressive fibrosing interstitial lung disease, *PSL* prednisolone, *PM/DM* polymyositis/dermatomyositis, *SjS* Sjögren’s syndrome, *SSc* systemic sclerosis, *Tac* tacrolimus, *UIP* usual interstitial pneumonia.

Data are expressed as medians (interquartile range) unless otherwise stated. Differences between two groups were evaluated by the Mann–Whitney U test and quantitative differences were tested by the Chi-squared test for goodness of fit or by Fisher’s exact test.

### Treatment at bronchoscopy and during FVC measurements

Of the 18 patients whose lung functions were compared 6 months before and at the time of bronchoscopy, 2 CTD-ILD patients had already been treated with corticosteroids or a combination of corticosteroids and tacrolimus; the other 16 patients were untreated. On the other hand, of the 12 patients whose lung functions were compared at the time of and 6 months after bronchoscopy, 9 patients were treated with corticosteroids alone (n = 3), a combination of corticosteroid and tacrolimus (n = 1), antigen avoidance (n = 4), or nintedanib (n = 1); the other 3 patients were untreated. Overall, 19 patients (63.3%) were untreated during FVC measurements, and 11 patients were treated with corticosteroids alone (n = 4), a combination of corticosteroid and tacrolimus (n = 2), antigen avoidance (n = 4), or nintedanib (n = 1). Only 1 of the 4 IPF patients with non-PF-ILDs was started on nintedanib after bronchoscopy, and the relative decline in %FVC in the 6 months after nintedanib treatment was 0.5%. The other 8 IPF patients were untreated during the observation period, but 2 of them were started on nintedanib after confirmation of PF-ILDs. There was no difference in the treatment during FVC measurements between PF-ILDs and non-PF-ILDs (*p* = 0.762, Table 2).

### Clinical importance of variables for FVC improvement in fibrotic ILD patients

Fibrotic ILD patients were divided into patients with decreased %FVC and those with improved FVC over 6 months in order to identify indicators for patients with improved FVC. Patients with decreased %FVC included 7 patients with a slightly decreased %FVC less than 5% over 6 months. Fibrotic ILD patients with improved %FVC had an 8.8% (IQR 16.1%) increase in %FVC over 6 months compared to those with worse %FVC (− 10.4%, IQR − 12.2%, *p* < 0.001, Supplementary Table S1). There were no differences in age, height, weight, BMI, sex, smoking status, serum KL-6 level, serum LDH level, diagnosis of ILD, chest HRCT pattern, %FVC predicted at diagnostic bronchoscopy, treatment during FVC measurements, and prevalence of PF-ILDs between patients with improved %FVC and those with worse %FVC. Only the proportion of lymphocytes in BALF was significantly increased in patients with improved %FVC.

### Cellular profiles of BALF and peripheral blood in patients with non-PF-ILDs and PF-ILDs

There were no differences in total cell counts, proportions of macrophages, or CD4/CD8 ratios in BALF (Table 3). The proportions of eosinophils and neutrophils tended to be higher in patients with PF-ILDs than with non-PF-ILDs, but the differences were not significant. The proportion of lymphocytes in BALF, an indicator used in clinical practice to predict response to corticosteroids, was significantly lower in patients with PF-ILDs than in those with non-PF-ILDs (23.8% vs 9.8%, *p* = 0.043, Table 3, Fig. 2D). On the other hand, the proportion of Th2 cells (CCR6^−^CXCR3^−^CD4^+^ T cells) among conventional CD4^+^ T cells in BALF, but not in peripheral blood, was significantly higher in PF-ILDs than in non-PF-ILDs (42.0% vs 9.0%, *p* = 0.001, Table 3, Fig. 2E). Details of disease progression and the proportions of Th2 cells in BALF of patients with underlying diseases are shown in supplementary Table S2. The prevalence of an increased proportion of Th2 cells above a median of 14.9% was higher in patients with PF-ILDs (n = 11/13, 84.6%) than in patients with non-PF-ILDs (n = 4/17, 23.5%, P < 0.005). Two CTD-ILD cases with non-PF-ILDs had a significantly increased proportion of Th2 cells in BALF (62.7% and 50.7%). Nine of 11 untreated PF-ILD patients (81.8%) had an increased proportion of Th2 cells in BALF above the median of 14.9%.

**Table 3:** Cellular profiles of BALF and peripheral blood in patients with non-PF-ILDs and PF-ILDs.

	Non-PF-ILD n = 17	PF-ILD n = 13	P value
**BALF cellular profile**			
Total cell count (/ × 10^5^ ml)	3.02 (1.09)	3.07 (1.45)	0.621
Lymphocytes (%)	23.8 (20.8)	9.8 (5.0)	0.043
Macrophages (%)	73.8 (21.6)	73.6 (31.5)	0.805
Eosinophils (%)	1.3 (2.2)	2.8 (1.7)	0.094
Neutrophils (%)	3.3 (3.2)	7.5 (17.8)	0.072
CD4/CD8 ratio	1.37 (2.04)	1.93 (3.68)	0.408
Th2 cells among conventional CD4^+^ T cells in BALF (%)	9.0 (7.4)	42.0 (24.2)	0.001
Th2 cells among conventional CD4^+^ T cells in peripheral blood (%)	35.1 (17.0)	37.9 (25.2)	0.263

*BALF* bronchoalveolar lavage fluid, *PF-ILD* progressive fibrosing interstitial lung disease, *Th2* T helper type 2.

Data are expressed as medians (interquartile range) unless otherwise stated. Differences between two groups were evaluated by the Mann–Whitney U test.

### Correlations between various variables and relative change of %FVC over 6 months in patients with fibrotic ILDs

**Figure 3:**
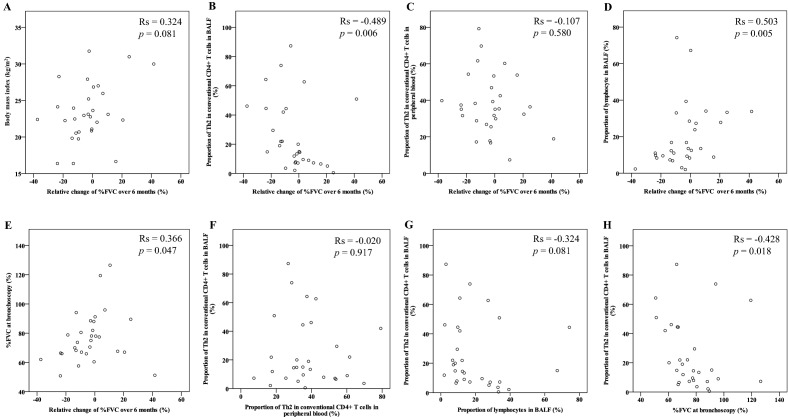
Correlation between various variables and relative change of %FVC over 6 months in patients with fibrotic interstitial lung diseases (ILDs). Body mass index (**A**), the proportion of Th2 cells among conventional CD4^+^ T cells in BALF (**B**) or that in peripheral blood (**C**), lymphocytes in BALF (**D**), or %FVC at bronchoscopy (**E**) vs relative change of %FVC predicted over 6 months. The proportion of Th2 cells in peripheral blood vs that in BALF (**F**). The proportion of lymphocytes or Th2 cells in BALF (**G**). The proportion of Th2 cells in BALF vs %FVC at bronchoscopy (**H**). The correlation coefficients between two variables were calculated using Spearman's rank correlation coefficient (Rs). BALF and peripheral blood samples were obtained from 30 and 29 patients with fibrotic ILDs, respectively. *BALF* bronchoalveolar lavage fluid, *FVC* forced vital capacity, *Th2 cell* T helper type 2 cell.

The BMI was not correlated with relative change of %FVC (Fig. 3A). The proportion of Th2 cells in BALF was inversely correlated with relative change of %FVC (Rs = − 0.489, *p* = 0.006, Fig. 3B), but this difference was not observed in Th2 cells in peripheral blood (Fig. 3C). The proportion of lymphocytes in BALF was correlated with relative change of %FVC (Rs = 0.503, *p* = 0.005, Fig. 3D) and %FVC at diagnostic bronchoscopy (Rs = 0.366, *p* = 0.047, Fig. 3E). The proportion of Th2 cells in BALF was not correlated with that in peripheral blood (Fig. 3F). The proportion of lymphocytes in BALF tended to be correlated, but not significantly, with the proportion of Th2 cells in BALF (Rs = − 0.324, *p* = 0.081, Fig. 3G). %FVC at diagnostic bronchoscopy was inversely correlated with the proportion of Th2 cells in BALF (Rs = − 0.142, *p* = 0.428, Fig. 3H).

Of the significantly different variables between PF-ILDs and non-PF-ILDs, multivariate analysis with binominal logistic regression showed that the proportion of Th2 cells in BALF was the only indicator associated with PF-ILD phenotype (Table 4). These results suggest that the Th2 subset in the lung, but not in peripheral blood, is associated with the progression of pulmonary fibrosis.

**Table 4:** Multivariate analysis by binominal logistic regression for determining PF-ILDs.

	Odds ratio	95% confidence interval	P value
Age (years)	0.962	0.807–1.147	0.666
Female sex	0.093	0.003–3.176	0.188
Body mass index (kg/m^2^)	0.639	0.376–1.085	0.097
Proportion of Th2 cells among conventional CD4^+^ T cells in BALF (%)	1.098	1.018–1.185	0.016
Proportion of lymphocytes in BALF (%)	0.962	0.878–1.054	0.402
%FVC predicted at BF (%)	0.942	0.869–1.021	0.146

*BALF* bronchoalveolar lavage fluid, *BF* bronchoscopy, *FVC* forced vital capacity; PF-ILD.

### Longitudinal changes in Th cell subsets of BALF and chest HRCT in an untreated IPF patient

**Figure 4:**
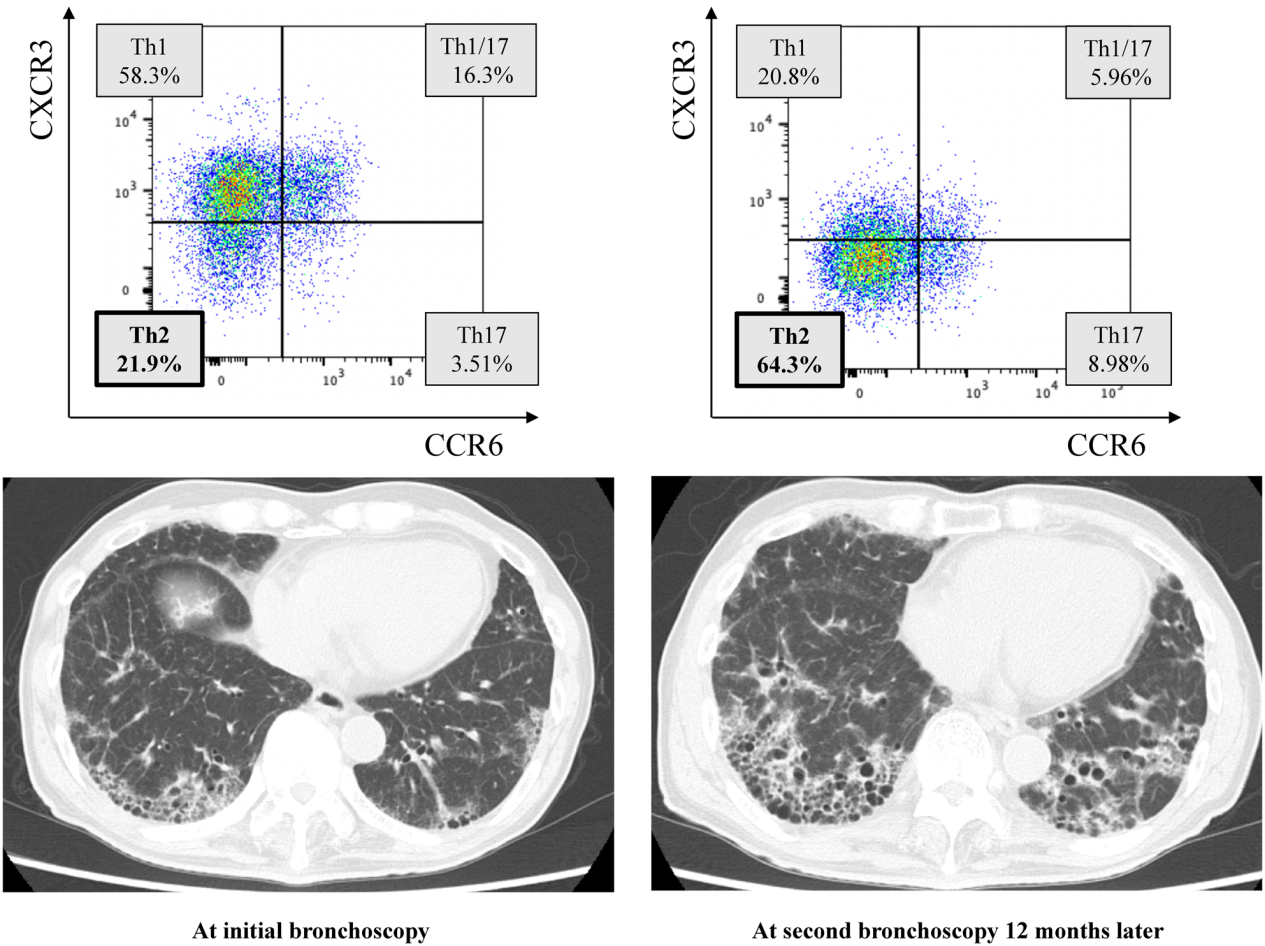
Longitudinal changes in Th cell subsets of BALF and chest HRCT images in an untreated IPF patients with PF-ILD phenotype. The proportion of Th2 cells in BALF had increased to 21.9% at initial bronchoscopy (**A**), but further increased to 64.3% at second bronchoscopy 12 months later (**B**), when chest HRCT showed the progression of pulmonary fibrosis. *BALF* bronchoalveolar lavage fluid, *FVC* forced vital capacity, *HRCT* high-resolution computed tomography, *IPF* idiopathic pulmonary fibrosis, *PF-ILD* progressive fibrosing interstitial lung disease.

BALF and chest HRCT were evaluated twice at 1-year intervals in an untreated IPF patient with PF-ILD phenotype (Fig. 4). The proportion of Th2 cells in BALF on the first bronchoscopy had increased to 21.9%, but it increased further to 64.3% on the second bronchoscopy 12 months later, when chest HRCT showed progression of pulmonary fibrosis. %FVC decreased from 68.2 to 50.8% in 1 year, and the relative %FVC decline from 6 months earlier was 13.0% and 23.8%, respectively. The proportion of lymphocytes in BALF was 12.3% and 11.0%, respectively. This case supports the importance of increasing Th2 cells in BALF for the disease progression of fibrotic ILDs.

## Discussion

The cellular profiles of BALF and peripheral blood were evaluated in fibrotic ILD patients. The present study found that PF-ILDs accounted for 43.3% of fibrotic ILDs with a 13.0% reduction in relative %FVC over 6 months. The proportion of Th2 cells (CCR6^−^CXCR3^−^CD4^+^ T cells) among conventional CD4^+^ T cells in BALF was significantly increased in PF-ILD patients compared with non-PF-ILD patients and inversely correlated with relative %FVC change. On the other hand, the proportion of lymphocytes in BALF was significantly increased in non-PF-ILD patients compared to PF-ILD patients and correlated with relative %FVC change. Multivariate analysis found that the proportion of Th2 cells in BALF was the only predictor of PF-ILDs. The increased proportion of Th2 cells in BALF may help to identify PF-ILDs and provide early intervention to improve the prognosis of these patients.

The present study found that 63.3% of fibrotic ILD patients were untreated during follow-up in clinical practice, and 43.3% of fibrotic ILD patients met the criteria of PF-ILDs and had relative %FVC change of − 13.0% over 6 months, which was a significantly greater decline than that of 0.6% in non-PF-ILD patients (Table 2). These data suggest that about half of fibrotic ILD patients had progressive deteriorations in lung function during follow-up, and these patients should have been treated before progression. Currently, antifibrotic agents such as pirfenidone and nintedanib have been reported to be effective in reducing lung function decline in PF-ILDs^5–7^. The timing of the introduction of antifibrotic agents in clinical practice depends on the individual judgment of the attending physicians, and many believe that it is best to start as early as possible if the disease has progressive fibrosing features. Since poorer performance status is the most common cause of the discontinuation of treatment, it is important to start antifibrotic drugs early, before performance status worsens^15^.

There have been no good indicators other than a decline in lung function and/or fibrotic changes on chest HRCT to determine PF-ILDs. There was no difference in serum KL-6 levels, diagnosis of ILD, or chest HRCT pattern between PF-ILD patients and non-PF-ILD patients at bronchoscopy. Since early diagnosis of PF-ILDs is an unmet need, the present data demonstrating for the first time that an increased proportion of Th2 cells in BALF at diagnosis is a possible predictor to identify PF-ILDs in patients with fibrotic ILDs are unprecedented and clinically important. These patients can be good candidates for antifibrotic treatment, whereas patients with an increased proportion of lymphocytes in BALF may be good candidates for anti-inflammatory therapy. Although a high proportion of Th2 cells can be affected by underlying diseases, especially CTD, it is important to determine the treatment strategy based on the combination with the proportion of Th2 cells and lymphocytes in BALF at diagnosis.

It should be noted that the proportion of Th2 cells in BALF, but not of eosinophils or neutrophils in BALF, was significantly higher in PF-ILD patients than in non-PF-ILD patients in the present study (Table 3). Previous reports have demonstrated that an increase in eosinophils or neutrophils in BALF is an indicator associated with the progression of ILDs^16–20^. Since the development, differentiation, activation, survival, and recruitment of eosinophils are associated with a Th2 cytokine, interleukin-5 (IL-5), an increase in eosinophils in BALF may reflect activation of the Th2 pathway in the lungs. Although the mechanism of lung fibrosis in association with Th2 cells was not clarified in the present study, several reports have demonstrated the relationship between pulmonary fibrosis and the Th2 pathway. Periostin, induced by the Th2 cytokines IL-4 and IL-13, has been reported to be involved in pulmonary fibrosis with crosstalk of transforming growth factor-β^21,22^, and serum periostin could be a good predictor of decreased lung function and poor prognosis^23–26^. Moreover, lines of evidence indicate that M2-like macrophages, known to be induced by Th2 cytokines, are profoundly involved in the pathogenesis of fibrosis^27–29^.

The present study also showed that the proportion of lymphocytes was significantly higher in non-PF-ILD patients than in PF-ILD patients (Table 3, Fig. 2D) and was correlated with relative %FVC change (Fig. 3D). Lymphocytosis in BALF is an important predictor of responsiveness to corticosteroids^16,17,30,31^. The present data confirmed that an increased proportion of lymphocytes in BALF was the only important difference in improving lung function in fibrotic ILD patients, with 18 of 21 (85.7%) patients being treated with corticosteroids and/or immunosuppressants in CTD-ILD or corticosteroids and/or antigen avoidance in fibrotic HP after evaluation of BALF cell differentiation. However, the proportion of lymphocytes in BALF did not predict PF-ILDs. Taken together with the results of lymphocytes in BALF for a good indicator of response to corticosteroids and/or immunosuppressants and Th2 cells in BALF for predicting PF-ILDs, analysis of BALF cellular profiles is very important for proper treatment of ILD patients, and bronchoscopy should be considered.

The BMI and %FVC predicted at bronchoscopy were significantly lower in patients with PF-ILDs than in those with non-PF-ILDs, although the difference in the median BMI was only 1.4 kg/m^2^. Two CTD-ILD cases with non-PF-ILDs had BMIs greater than 30 kg/m^2^. Two IPF patients with PF-ILDs and one CTD-ILD case with non-PF-ILD had a BMI less than 17.5 kg/m^2^. There are several possible effects of BMI on fibrotic ILDs. First, large amounts of pericardial and abdominal visceral adipose tissue have been reported to be associated with a higher prevalence of interstitial lung abnormalities associated with gastroesophageal reflux and a lower FVC^32^. Second, the association between obesity and immune balance may affect the pathogenesis of various lung diseases^33^. Third, both low BMI and weight loss have been reported to be independently associated with increased 1-year mortality in fibrotic ILD^34^. Although it is unclear whether the weight loss was caused by loss of adipose tissue or muscle mass, or whether poor nutritional status or reduced activity was involved in the progression of lung fibrosis, nutritional support is also important in the treatment strategy for PF-ILDs.

There are no standard criteria for PF-ILDs. One paper has proposed any of the following criteria within 24 months as evidence of disease progression: a relative decline of 10% or greater in %FVC predicted; a relative decline of 15% or greater in diffusing capacity of the lung for carbon monoxide; worsening symptoms; or radiological appearance accompanied by a 5% or greater but less than 10% relative decrease in %FVC predicted^7^. However, another study of pirfenidone used a criterion of more than a 5% absolute decrease in %FVC predicted for at least 6 months^35^.

The present research has several limitations. First, a small number of study subjects in a single center for respiratory diseases were included. Even though a small number of subjects were included, the present study clearly showed that the proportion of Th2 cells in BALF was the only indicator of PF-ILDs. Further large, prospective studies are needed to validate the higher proportion of Th2 cells in BALF as a biomarker to predict PF-ILDs. Second, it was not possible to incorporate the data for diffusing capacity of the lung for carbon monoxide, 6-min walking distance, and quality of life scores into this study, because they were not available for all patients. Third, a direct mechanism for how Th2 cells are involved in the progression of pulmonary fibrosis cannot be provided. Functional analysis with cytokines and transcription factors is needed for Th2 cells, which are involved in fibrosis progression. Fourth, the underlying diagnosis of ILD was heterogeneous, including IPF, fibrotic HP, and CTD-ILD. Different backgrounds of the diseases may have their own specific immunological responses, which may have affected the study results. It should be noted that an increased proportion of Th2 cells in BALF of fibrotic ILDs will not allow one to reach any conclusion regarding the underlying disease within PF-ILDs, because PF-ILDs had a high proportion of Th2 cells in BALF regardless of the underlying disease. Recent studies have shown that a certain number of PF-ILD cases are present regardless of the underlying diseases^36^, and nintedanib is effective for slowing lung function decline in such heterogeneous PF-ILDs^37^. Fifth, the effect of antifibrotic drugs on Th2-high ILD patients could not be investigated in the present study, because only one IPF patient was treated with nintedanib during FVC measurement, and two more were started on it after evaluation. Further large prospective studies are needed to evaluate the efficacy of antifibrotic drugs in such PF-ILD patients with increased Th2 cells in BALF.

In conclusion, the predominance of Th2 cells in BALF is an indicator of a progressively greater decrease of lung function in fibrotic-ILDs, and these patients can be good candidates for antifibrotic therapy.

## Supplementary Information

Supplementary Information.

## Data Availability

The supplementary files for the detailed procedure of flowcytometry and Supplementary Table S1 and S2 are accessible online. The datasets used and/or analyzed during the current study are available from the corresponding author on reasonable request.
