# Supplementary material for: Lung CCR6−CXCR3− type 2 helper T cells as an indicator of progressive fibrosing interstitial lung diseases
Source: Sci Rep. 2022 Nov 15;12:19577. doi: 10.1038/s41598-022-24011-0 (PMC9666512; doi:10.1038/s41598-022-24011-0)
Supplement: Supplementary file 1 — Supplementary Information. [file 41598_2022_24011_MOESM1_ESM.docx]

**Lung CCR6^-^CXCR3^-^ Th2 cells as an indicator of progressive fibrosing interstitial lung diseases**

Tsukie Kin Tsukuda^1^, Hiroshi Ohnishi^1, *^, Minoru Fujimoto^2, 3^, Yu Nakatani^1^,

Kazufumi Takamatsu^1^, Tetsuji Naka^2, 3^, and Akihito Yokoyama^1^

^1^ Department of Respiratory Medicine and Allergology, Kochi Medical School, Kochi University, Nankoku, Japan

^2^ Center for the Intractable Immune Disease, Kochi Medical School, Kochi University, Nankoku, Japan

^3^ Division of Allergy and Rheumatology, Department of Internal Medicine, School of Medicine, Iwate Medical University, Morioka, Japan

**Supplementary information**

**Supplementary Methods**

**Preparation of bronchoalveolar lavage fluid (BALF) for flow cytometry**

Within 1  h following the bronchoscopy, BALF (stored at 4 °C) was centrifuged (300 *g* for 10 minutes), and supernatant was aspirated. BAL cells were resuspended with sterile MACS buffer (PBS, pH 7.2; 2 mM EDTA; 0.5% bovine serum albumin). Erythrocytes were lysed through incubation with ammonium-chloride-potassium (ACK) Lysing Buffer and resuspended with Dulbecco’s phosphate-buffered saline (D-PBS). Cell viability was assessed by Trypan Blue staining. For each staining set, 1 ml of suspension was placed in 2-ml Eppendorf tubes. BAL cells were spun down (10 min at 300 *g*), and then supernatant was aspirated and resuspended with MACS Buffer. The tubes were each stained with anti-human antibodies and left for 20 min at 4 °C in the dark and then spun down (5 min at 300 *g*) and resuspended. Then, 300 µl of MACS Buffer were added into the tubes, and the cell suspension was filtered through to 5-ml round-bottom tubes and stored at 4 °C in the dark until measurement that day. BALF samples contained a median total cell count of 3.05 x 10^5^/ml with an interquartile range of 1.13 x 10^5^/ml. We have compared the effects of a total of 10 colors staining between a staining set using 8 antibodies and a staining set using 2 additional antibody on 10 BALF samples and 20 blood samples from ILD patients including some patients entered in this study. We did not find any significant differences between the results of the two sets of analysis.

**Preparation of peripheral blood mononuclear cells for flow cytometry**

Peripheral blood samples from study subjects were collected in EDTA tubes. Then, 100 µl were placed in 2-ml safe-rock tubes and stained with combinations of the anti-human antibodies and left for 20 min at 4 °C in the dark. To fix and hemolyze the cells, FACS Lysing Solution (FACS lyse, Becton Dickinson Immunocytometry Systems [BDIS]) was added into tubes for 15 min at room temperature. Flow-Count fluorospheres (Beckman Coulter, Brea, CA) were used for measurement of absolute cell counts. The suspension supernatant was aspirated and resuspended with MACS Buffer and centrifuged (500 *g* for 5 minutes) again, and the supernatant was aspirated. Cells were suspended with MACS Buffer, and the cell suspension was filtered through to 5-ml round-bottom tubes and stored at 4 °C in the dark until measurement.

**Supplementary Table S1. Patients’ characteristics between fibrotic ILDs with improved %FVC and worse %FVC**

|  | **Improved %FVC** (n=10)  (33.3%) | **Worse %FVC** (n=20)  (66.7%) | P value |
| --- | --- | --- | --- |
| Age (y) | 74.5 (12.2) | 72 (7.0) | 0.428 |
| Height (cm) | 155.5 (10.3) | 160.0 (9.5) | 0.481 |
| Weight (kg) | 61.3 (17.0) | 56.1 (15.7) | 0.454 |
| Body mass index (kg/m^2^) | 24.8 (4.4) | 22.4 (3.3) | 0.129 |
| Sex, male [n (%)] | 6 (60.0) | 14 (70.0) | 0.690 |
| Smoking status (n)  Never/former/current | 1/8/1 | 5/15/0 | 0.306 |
| Serum KL-6 level (U/mL) | 1107 (1131) | 939 (820) | 0.475 |
| Serum LDH level (U/L) | 266 (109) | 236 (50) | 0.428 |
| Diagnosis of ILD (n)  IPF  Fibrotic HP  CTD-ILD  　SSc  　PM/DM  　SjS  　MCTD | 2  4  4  1  2  0  1 | 7  7  6  3  2  1  0 | 0.792 |
| Chest HRCT pattern (n)  　f-NSIP  　UIP | 3  7 | 3  17 | 0.372 |
| %FVC predicted at diagnostic BF (%) | 83.8 (24.7) | 70.2 (13.2) | 0.078 |
| Relative %FVC change over 6 months (%) | 8.8 (16.1) | -10.4 (12.2) | <0.001 |
| Treatment during FVC  measurements  None  PSL  PSL + Tac  Antigen avoidance  Nintedanib | 6  2  1  1  0 | 13  2  1  3  1 | 0.946 |
| BALF cellular profile |  |  |  |
| Total cell counts (/×10^5^ ml) | 3.35 (1.50) | 2.96 (1.03) | 0.588 |
| Lymphocytes (%) | 27.6 (17.6) | 10.4 (8.8) | 0.018 |
| Macrophages (%) | 66.9 (22.0) | 76.0 (27.8) | 0.169 |
| Eosinophils (%) | 1.6 (4.5) | 2.4 (2.48) | 0.947 |
| Neutrophils (%) | 2.8 (6.5) | 5.3 (8.3) | 0.179 |
| CD4/CD8 ratio | 1.29 (2.53) | 1.63 (2.73) | 0.355 |
| Th2 cells among conventional CD4^+^ T cells in BALF (%) | 9.3 (8.2) | 21.0 (33.5) | 0.147 |
| Th2 cells among conventional CD4^+^ T cells in peripheral blood (%) | 35.2 (10.5) | 37.4 (22.3) | 0.748 |

BALF = bronchoalveolar lavage fluid; BF = bronchoscopy; CTD = connective tissue disease; f-NSIP = fibrotic nonspecific interstitial pneumonia; FVC = forced vital capacity; HP = hypersensitivity pneumonitis; HRCT = high-resolution computed tomography; ILD = interstitial lung disease; IPF = idiopathic pulmonary fibrosis; KL-6 = Krebs von den Lungen-6; LDH = lactate dehydrogenase; MCTD = mixed connective tissue disease; PF-ILD = progressive fibrosing interstitial lung disease; PM/DM = polymyositis/dermatomyositis; SjS = Sjögren’s syndrome; SSc = systemic sclerosis; Th2 cell= T helper type 2 cell; UIP = usual interstitial pneumonia

Data are expressed as medians (interquartile range) unless otherwise stated. Differences between two groups were evaluated by the Mann-Whitney U test and quantitative differences were tested by the Chi-squared test for goodness of fit or by Fisher’s exact test.

**Supplementary Table S2. Disease progression and the proportion of Th2 cells in BALF of fibrotic ILD patients with underlying diseases**

| Diagnosis | Progression | Th2 cells in BALF | | Relative %FVC change (%) | | Treatment at the time of BF |
| --- | --- | --- | --- | --- | --- | --- |
|  |  | The proportion of Th2 cells | High or low divided by median |  |  |  |
| IPF | PF-ILD | 64.3 | high | -23.84 | none | |
|  |  | 44.5 | high | -23.68 | none | |
|  |  | 29.5 | high | -18.68 | none | |
|  |  | 22.0 | high | -12.05 | none | |
|  |  | 21.9 | high | -13.01 | none | |
|  | Non-PF-ILD | 20.0 | high | -0.50 | none | |
|  |  | 14.5 | low | 0.65 | none | |
|  |  | 13.4 | low | -1.44 | none | |
|  |  | 9.0 | low | 7.03 | none | |
| HP | PF-ILD | 73.9 | high | -12.87 | none | |
|  |  | 46.1 | high | -37.42 | PSL | |
|  |  | 44.4 | high | -9.21 | none | |
|  |  | 19.0 | high | -13.86 | none | |
|  |  | 3.6 | low | -9.55 | none | |
|  | Non-PF-ILD | 15.0 | high | 0.22 | none | |
|  |  | 11.9 | low | -3.40 | none | |
|  |  | 9.5 | low | 3.06 | none | |
|  |  | 7.9 | low | -2.30 | none | |
|  |  | 7.3 | low | 10.58 | none | |
|  |  | 5.1 | low | 20.60 | none | |
| CTD-ILD | PF-ILD | 87.3 | high | -5.86 | PSL+Tac | |
|  |  | 42.0 | high | -11.25 | none | |
|  |  | 14.8 | low | -22.81 | none | |
|  | Non-PF-ILD | 62.7 | high | 3.85 | none | |
|  |  | 50.9 | high | 41.60 | none | |
|  |  | 7.3 | low | -2.67 | none | |
|  |  | 7.2 | low | -0.68 | PSL | |
|  |  | 6.5 | low | 15.83 | none | |
|  |  | 2.1 | low | -2.94 | none | |
|  |  | 0.5 | low | 24.92 | none | |

BALF = bronchoalveolar lavage fluid; BF = bronchoscopy; CTD-ILD = connective tissue disease-associated interstitial lung disease; FVC = forced vital capacity; HP = hypersensitivity pneumonitis; IPF = idiopathic pulmonary fibrosis; PF-ILD = progressive fibrosing interstitial lung disease; PSL = prednisolone; Tac = tacrolimus; Th2 cell= T helper type 2 cell
